# Exploring EEG Features in Cross-Subject Emotion Recognition

**DOI:** 10.3389/fnins.2018.00162

**Published:** 2018-03-19

**Authors:** Xiang Li, Dawei Song, Peng Zhang, Yazhou Zhang, Yuexian Hou, Bin Hu

**Affiliations:** ^1^Tianjin Key Laboratory of Cognitive Computing and Application, Tianjin University, Tianjin, China; ^2^School of Computer Science and Technology, Beijing Institute of Technology, Beijing, China; ^3^School of Computing and Communications, The Open University, Milton Keynes, United Kingdom; ^4^School of Information Science and Engineering, Lanzhou University, Lanzhou, China

**Keywords:** EEG, emotion recognition, feature engineering, DEAP dataset, SEED dataset

## Abstract

Recognizing cross-subject emotions based on brain imaging data, e.g., EEG, has always been difficult due to the poor generalizability of features across subjects. Thus, systematically exploring the ability of different EEG features to identify emotional information across subjects is crucial. Prior related work has explored this question based only on one or two kinds of features, and different findings and conclusions have been presented. In this work, we aim at a more comprehensive investigation on this question with a wider range of feature types, including 18 kinds of linear and non-linear EEG features. The effectiveness of these features was examined on two publicly accessible datasets, namely, the dataset for emotion analysis using physiological signals (DEAP) and the SJTU emotion EEG dataset (SEED). We adopted the support vector machine (SVM) approach and the “leave-one-subject-out” verification strategy to evaluate recognition performance. Using automatic feature selection methods, the highest mean recognition accuracy of 59.06% (AUC = 0.605) on the DEAP dataset and of 83.33% (AUC = 0.904) on the SEED dataset were reached. Furthermore, using manually operated feature selection on the SEED dataset, we explored the importance of different EEG features in cross-subject emotion recognition from multiple perspectives, including different channels, brain regions, rhythms, and feature types. For example, we found that the Hjorth parameter of mobility in the beta rhythm achieved the best mean recognition accuracy compared to the other features. Through a pilot correlation analysis, we further examined the highly correlated features, for a better understanding of the implications hidden in those features that allow for differentiating cross-subject emotions. Various remarkable observations have been made. The results of this paper validate the possibility of exploring robust EEG features in cross-subject emotion recognition.

## 1. Introduction

Emotion recognition as an emerging research direction has attracted increasing attention from different fields and is promising for many application domains. For example, in human-computer interaction (HCI), recognized user emotion can be utilized as a kind of feedback to provide better content to enhance the user experiences in e-learning, computer games, and information retrieval (Mao and Li, 2009; Chanel et al., 2011; Moshfeghi, 2012). Moreover, psychologists have verified the important roles that emotion plays in human health. Difficulties in the regulation of negative emotions may cause various mood disorders, such as stress and depression (Gross and Muñoz, 1995), which may influence people's health (O'Leary, 1990). Hence, emotion recognition techniques also contribute to developing e-services for mental health monitoring. In particular, cross-subject emotion recognition (i.e., depression prediction based on a person's physiological data, with a classifier learnt from the training data from a group of patients who have been diagnosed as depression or not) has been considered an important task for its generality and wider applicability, compared with the intra-subject emotion recognition.

Electroencephalogram (EEG) measurements reflect the neural oscillations of the central nervous system (CNS) and are directly related to various higher-level cognitive processes (Ward, 2003), including emotion (Coan and Allen, 2004). EEG-based emotion recognition has shown a greater potential compared with the facial expression- and speech-based approaches, as the internal neural fluctuations cannot be deliberately concealed or controlled. However, a main issue confronted in this research area is how to improve the cross-subject recognition performance. The performances of current recognition systems are largely limited by the poor generalizability of the EEG features in reflecting emotional information across subjects. For example, Kim (2007) studied the bimodal data fusion method and utilized LDA to classify emotions. Using this method, the best obtained recognition accuracy on all three subjects' data was 55%, which was far lower than the best result of 92% obtained on a single subject's data. Zhu et al. (2015) adopted differential entropy as the emotion-related feature and the linear SVM as the classifier. The authors verified the recognition performance on intra-subject and cross-subject experimental settings respectively. The average recognition accuracy was 64.82% for cross-subject recognition tasks, which was also far lower than the results of 90.97% obtained in the intra-subject settings.

In the literature, there has been some related work that attempted to tackle this problem and to identify robust EEG features in cross-subject emotion recognition. For example, Li and Lu (2009) examined the recognition performance using ERD/ERS features extracted from different frequency bands and found that 43.5–94.5 Hz, the higher gamma band, was the optimal frequency band related to happiness and sadness. Lin et al. (2010) extracted DASM features and summarized the top 30 subject-independent features by measuring the ratio of between- and within-class variance, and found that the frontal and parietal electrode pairs were the most informative on emotional states. However, no significant difference between different frequency bands was observed in this work. Soleymani et al. (2012) performed cross-subject emotion recognition tasks on EEG and eye gaze data. The power spectral density (PSD) for EEGs was extracted. The most discriminative features for arousal were in the alpha band of the occipital electrodes, while those for valence were in the beta and gamma bands of the temporal electrodes. Kortelainen and Seppänen (2013) extracted the PSD from different frequency bands, and the best cross-subject classification rate for valence and arousal was obtained on the feature subset in the 1–32 Hz band. Zheng et al. (2016) focused on finding stable neural EEG patterns across subjects and sessions for emotion recognition. The authors found that EEGs in lateral temporal areas were activated more for positive emotions than negative emotions in the beta and gamma bands and that subject-independent EEG features stemmed mostly from those brain areas and frequency bands.

In the aforementioned existing work, however, only few kinds of features were examined and why those robust features contribute to cross-subject emotion recognition was not studied. Hence, in this work, we aim at a more comprehensive and systematic exploration of a wider range of EEG features. Specifically, we extracted nine kinds of time-frequency domain features and nine kinds of dynamical system features from EEG measurements. Through automatic feature selection, e.g., recursive feature elimination (RFE), we verified the effectiveness and performance upper bounds of those features in cross-subject emotion recognition. Furthermore, through manual selection of features from different aspects, e.g., different EEG channels, we studied the importance of different aspects in cross-subject emotion recognition. We further conducted a correlation analysis to better understand the implications of those features for differentiating cross-subject emotions. The support vector machine (SVM), a state of the art classifier, was used in all the experiments.

## 2. Materials and methods

**Figure 1:**
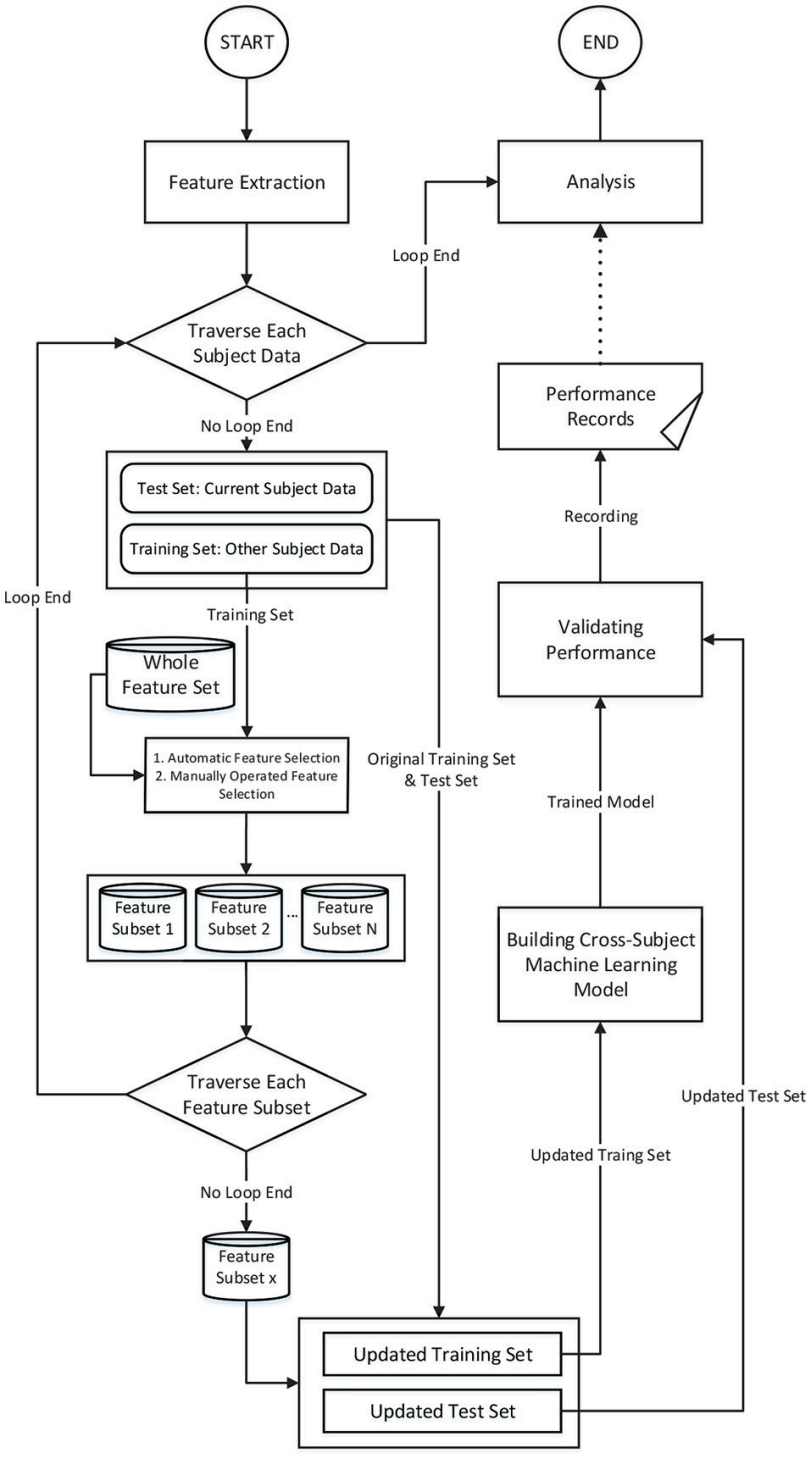
The feature engineering-based method and the procedure for verifying the performance of cross-subject emotion recognition.

The procedure of the proposed methodology is illustrated in Figure 1. We adopted a “leave-one-subject-out” verification strategy. Each time we left one subject's data out as the test set and adopted the other subjects' data as the training set. The feature selection was conducted on the training set, and then, the performance was evaluated on the test set. This procedure was iterated until each subject's data had been tested. This strategy can eliminate the risk of “overfitting.”

### 2.1. Experimental data

We conducted our analysis using two publicly accessible datasets, namely, DEAP (dataset for emotion analysis using physiological signals) (Koelstra et al., 2012) and SEED (SJTU emotion EEG dataset) (Zheng et al., 2016). DEAP includes 32-channel EEG data collected from 32 subjects (17 male, 27.2 ± 4.4 years). The subjects' emotions were induced through one-minute-long music video clips. After each stimulus, the subjects rated their emotional experience on a two-dimensional emotional scale proposed by Russell (1980). The two dimensions are arousal (ranging from relaxed to aroused) and valence (ranging from unpleasant to pleasant). The higher a specific rating is, the more intense the emotion is in a specific dimension. The SEED dataset contains 62-channel EEG data collected from 15 subjects (7 male, 23.27 ± 2.37 years), and each subject participated in the experiment three times. The subjects' emotions are induced through 15 film clips, and each film clip lasts for approximately 4 min. Three classes of emotions (positive, neutral, negative) are evaluated, and each class has five corresponding film clips. In this study, we utilized only the trials of positive and negative emotions to evaluate the features' ability to differentiate between these two emotions. For consistency with the DEAP dataset, we used the one-minute-long data extracted from the middle part of each trial in SEED.

### 2.2. Data preprocessing

#### 2.2.1. EEG preprocessing

**Figure 2:**
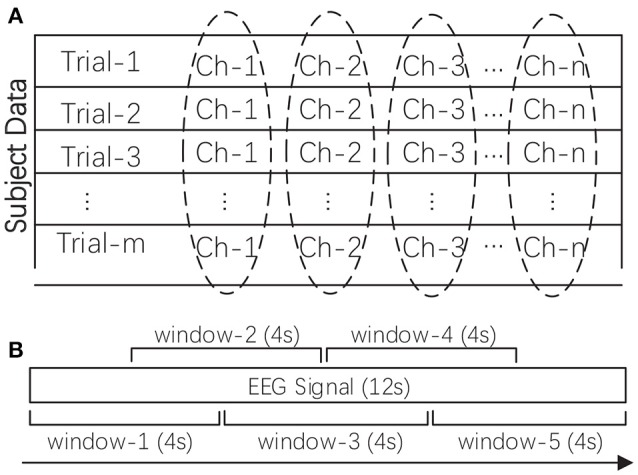
**(A)** The data normalization method for one subject's multi-channel signals. **(B)** The sliding window-based feature extraction method for one EEG signal (taking one 12-s signal as an example). The mean of the calculated values in all sliding windows was adopted as the feature.

As a kind of neurophysiological signal, EEG data are high dimensional and contain redundant and noisy information. In this work, after data acquisition, the raw data was firstly pre-processed, such as by removing the electrooculogram (EOG) and electromyogram (EMG) artifacts and downsampling the raw data to reduce the computational overhead in feature extraction. Two additional preprocessing procedures were needed before feature extraction, namely, rhythm extraction and data normalization. The multi-channel EEG is typically regarded as a reflection of brain rhythms. We first filtered out the four target rhythms, namely, the theta rhythm (4–7 Hz), alpha rhythm (8–15 Hz), beta rhythm (16–31 Hz), and gamma rhythm (>32 Hz). We attempted to investigate the importance of these different rhythms in reflecting subjects' emotions. We excluded the delta rhythm (<4 Hz), as this rhythm is traditionally regarded as being correlated only with sleep. The four target rhythms were extracted through a custom finite impulse response (FIR) bandpass filter with a Hanning window. Secondly, we conducted data normalization as shown in Figure 2A. The extracted rhythm data for each subject were normalized channel by channel across all the trials. This procedure helped to remove subject bias and to generate more comparable features between subjects while allowing the variability of different channels to be preserved.

#### 2.2.2. Label preprocessing

For DEAP, we divided the subject trials into two classes according to their corresponding ratings on the valence dimension. A rating higher than 5 indicated a positive class, whereas a rating lower than 5 indicated a negative class. Hence, for valence, the two classes were high valence (positive) and low valence (negative). For SEED, the trials have already been categorized into three emotional classes (positive, neutral, negative); hence, we do not need to perform label preprocessing. For consistency, we studied only the positive and negative samples in SEED. The emotion recognition capability was evaluated using binary classification tasks.

### 2.3. Feature extraction

In this work, we explored the robustness of a wider range of EEG features in cross-subject emotion recognition. Specifically, we extracted nine kinds of time-frequency domain features and nine kinds of dynamical system features from EEG measurements, as listed in Table 1. Extracting features based on some domain knowledge can provide a concise representation of the original data and materials. In this work, after preprocessing the data, we calculated the features for each of the four rhythms with a 4-s sliding window and a 2-s overlap, and then, the mean of the feature values extracted from those sliding windows was adopted as the trial's feature. The sliding window-based feature extraction methods are illustrated in Figure 2B. For DEAP, the number of features extracted for one trial is: ((9 + 9) × 32) × 4 = 2304. For SEED, the number of features extracted for one trial is: ((9 + 9) × 62) × 4 = 4464. All features were normalized before further analysis.

**Table 1:** This table lists the two main categories of EEG features that we extracted.

**Feature type**	**Extracted features**
Time-frequency domain features	1. Peak-Peak Mean. 2. Mean Square Value. 3. Variance.
	4. Hjorth Parameter: Activity. 5. Hjorth Parameter: Mobility.
	6. Hjorth Parameter: Complexity.
	7. Maximum Power Spectral Frequency.
	8. Maximum Power Spectral Density. 9. Power Sum.
Non-linear dynamical system features	10. Approximate Entropy. 11. C0 Complexity.
	12. Correlation Dimension. 13. Kolmogorov Entropy.
	14. Lyapunov Exponent. 15. Permutation Entropy.
	16. Singular Entropy. 17. Shannon Entropy. 18. Spectral Entropy.

*The features were extracted for four rhythms. For the DEAP dataset, the total number of features extracted for one trial is 2304. For the SEED dataset, the total number of features extracted for one trial is 4464*.

The details and reasons for selecting these candidate features are elaborated below:

#### 2.3.1. Time-frequency domain features

Nine kinds of features in the time and frequency domains of each signal were considered. The *peak-to-peak mean* is the arithmetic mean of the vertical length from the very top to the very bottom of the time series. The *mean squared value* is the arithmetic mean of the squares of the time series. *Variance* measures the degree of dispersion of the time series. After transforming the time series into the frequency domain through Fourier transform, we calculated the sum of the power spectral, and we further extracted the maximum power spectral density along with its corresponding frequency value. Three *Hjorth parameters* that can reflect characteristics of activity, mobility, and complexity were also extracted according to the work by Hjorth (1970): the *activity* parameter reflects the information of the signal power, the *mobility* parameter is an estimation of the mean frequency, and the *complexity* reflects the bandwidth and the change in frequency. The Hjorth parameters are considered suitable for analyzing non-stationary EEG signals.

#### 2.3.2. Non-linear dynamical system features

We also extracted nine kinds of features that can reflect the characteristics of non-linear dynamical systems. Researchers have found that human brain manifests many characteristics specifically belonging to non-linear and chaotic dynamical systems; thus, the EEG signal is inherently complex, non-linear, non-stationary, and random in nature (Stam, 2005; Sanei and Chambers, 2013). *Approximate entropy (ApEn)* is a non-linear measure of the regularity of a signal; the more regular a signal is, the smaller the ApEn will be (Pincus et al., 1991). *C0 Complexity* is adopted to measure the amount of the stochastic components, which assumes that a signal consists of a regular part and a stochastic part (Lu et al., 2008). *Correlation dimension* determines the number of dimensions (independent variables) that can describe the dynamics of the system and reflects the complexity of the process and the distribution of system states in the phase space (Khalili and Moradi, 2009). The *Lyapunov exponent* is used to measure the aperiodic dynamics of a chaotic system. This feature can capture the separation and evolution of the system's initial states in the phase space. The positive Lyapunov exponent indicates the chaos in the system (Übeyli, 2010). The *Kolmogorov entropy* is also a metric of the degree of chaos and measures the rate at which information is produced by the system as well as the rate at which information is lost by the system (Aftanas et al., 1997). Note that ApEn is closely related to Komolgorov entropy. The calculation of Komolgorov entropy is greatly influenced by the noise and dimensionality of the data. The complexity of neural activity can also be measured using the symbolic dynamic theory, in which a time series can be mapped to a symbolic sequence, from which the *permutation entropy (PE)* can be derived. The largest value of PE is 1, which indicates that the time series is completely random, while the smallest value of PE is 0, which indicates that the time series is completely regular (Li et al., 2007). *Singular spectrum entropy* is calculated by a singular value decomposition (SVD) of the trajectory, which is obtained by reconstructing the one-dimensional time series into a multi-dimensional phase space. This feature reflects the uncertainty and complexity of the energy distribution and is an indicator of event-related desynchronization (ERD) and event-related synchronization (ERS) (Zhang et al., 2009). *Shannon entropy* is a classical quantification of uncertainty and is frequently used to measure the degree of chaos in the EEG signal. *Power spectral entropy* is based on the Shannon entropy and measures the spectral complexity of the system. After the Fourier transform is performed, the signal is transformed into a power spectrum, and the information entropy of the power spectrum is called the power spectral entropy (Zhang et al., 2008).

### 2.4. Automatic feature selection

In this work, we first try to determine the upper bound of the performance of the proposed features. Hence, we choose to utilize some automatic feature selection techniques. Five different automatic feature selection methods were used to extract the most informative EEG features from the whole candidate set. Specifically, the whole features are re-ranked according to a pre-defined ranking criteria, e.g., based on the degree of correlation between the feature and the target class or based on the value of the feature weight, and then, the features above a pre-defined threshold are selected (Huang et al., 2006; Maldonado and Weber, 2008).

Two typical automatic feature selection techniques are the filter-based strategy and the wrapper-based strategy (Guyon and Elisseeff, 2003). The former is independent of any pattern recognition algorithm and filters out a specific number of features according to some statistical properties of the features. The classical filter-based strategy includes the chi-squared (χ^2^), mutual information, and *F*-test methods. The wrapper-based strategy, on the other hand, cooperates with a specific pattern recognition algorithm. A widely adopted wrapper-based strategy is the recursive feature elimination (RFE) method. We also considered a more efficient L1-norm penalty-based feature selection method, which has been widely used in recent years.

The details of these feature selection methods are elaborated as follows:

#### 2.4.1. Chi-squared-based feature selection (χ^2^)

The Chi-squared test is a classical statistical hypothesis test method for testing the independence of two variables or to investigate whether the distribution of one variable differs from that of another. This work is concerned with the former, formulated as below:

(1) $$\chi^{2}=\sum_{i=1}^{r}\sum_{j=1}^{c}\frac{{\left(O_{i,j}-E_{i,j}\right)}^{2}}{E_{i,j}},$$

where *r* and *c* are the number of categories in the two random variables, *O*_*i,j*_ is the number of observations of type *i, j*, and the *E*_*i,j*_ is the expected frequency of type *i, j*. In our work, a higher χ^2^ value indicates a higher correlation between a feature variable and the target classes.

#### 2.4.2. Mutual information-based feature selection (MI)

The mutual information metric is derived from probability theory and information theory. This metric is adopted to measure the mutual dependence (shared information) between the feature variables and the target classes. It is closely linked to the concept of entropy, which defines how much information is contained in a variable. Mutual information can be expressed as follows:

(2) $$\begin{array}{l} I(X;Y)=H(X)+H(Y)-H(X,Y)\\ \;=\sum_{x}P(x)\log\frac{1}{P(x)}+\sum_{y}P(y)\log\frac{1}{P(y)}\\ \;-\sum_{x,y}P(x,y)\log\frac{1}{P(x,y)}\\ \;=\sum_{x,y}P(x,y)\log\frac{p(x,y)}{P(x)p(y)}, \end{array}$$

where *H*(*X*) and *H*(*Y*) are the marginal entropy of *X* and *Y* respectively, and *H*(*X, Y*) is the joint entropy of *X* and *Y*.

#### 2.4.3. ANOVA *F*-value-based feature selection (AF)

F-test is a representative version of the analysis of variance (ANOVA). It is typically used to test whether the means of multiple populations are significantly different. In feature selection, ANOVA can measure the “F-ratio” of the between-class variance (as in Equation 4) over within-class variance (as in Equation 5). The “F-ratio” indicates the degree of class separation, as formulated in Equation (3). The higher a feature variable's F-ratio is, the better this feature is in differentiating different classes.

(3) $$F_{ratio}=\frac{\sigma_{between}^{2}}{\sigma_{within}^{2}}$$

where the between-class variance and within-class variance are:

(4) $$\sigma_{between}^{2}=\frac{\sum_{j=1}^{J}{\left({\bar{x}}_{j}-\bar{x}\right)}^{2}N_{j}}{J-1}$$

and

(5) $$\sigma_{within}^{2}=\frac{(\sum_{j=1}^{J}\sum_{i=1}^{N_{j}}{\left(x_{i,j}-\bar{x}\right)}^{2})-(\sum_{j=1}^{J}{\left({\bar{x}}_{j}-\bar{x}\right)}^{2}N_{j})}{N-J},$$

respectively. *J* is the number of classes, *N*_*j*_ is the number of measurements in the *j*th class, ${\bar{x}}_{j}$ is the mean of the *j*th class, $\bar{x}$ is the overall mean, and *x*_*i,j*_ is the *i*th measurement of the *j*th class.

#### 2.4.4. Recursive feature elimination (RFE)

As first introduced in Guyon et al. (2002) for gene selection, RFE is a wrapper-based method that judges the importance of features using an external machine learning algorithm. It adopts a sequential backward elimination strategy. First, the algorithm is trained on the initial whole set of features and assigns weight to each of the features. Then, a pre-defined number of features with the lowest-ranking absolute weights are pruned from the current feature set. This procedure recursively repeats for several steps until the desired number of selected features is reached. The pseudo code of the RFE is illustrated below in Algorithm 1. In this work, we used SVM with a linear kernel as the ranking method, in which the RFE utilized ||*w*|| as the ranking criteria for the importance of the features.

**Algorithm 1:** Pseudo Code for Recursive Feature Elimination (RFE) Algorithm

**Input:**
Training set: *T*
Feature set: *F* = {*f*_1_, *f*_2_, …, *f*_*p*_}
Ranking method: *M*(*T, F*)
Desired feature number: *q*
Number of feature to eliminate in each step: *k*
**Output:**
Final ranking feature set: *R* = {*f*_*r*1_, *f*_*r*2_, …, *f*_*rp*_}
Final selected feature set: *F* = {*f*_1_, *f*_2_, …, *f*_*q*_}
1 **Initialization;**
2 Steps: S = (p-q)/k;
3 **for** *i* = 1 → *S* **do**
$\begin{array}{cc} \begin{array}{l} 4\\ 5\\ 6\\ 7 \end{array} & |\begin{array}{l} \text{ Rank set }F\text{ according to }M\text{(}T,F\text{);}\\ \;L_{k}\leftarrow \text{Last ranked }k\text{ features in }F;\;\\ \;R[p-i*k+1:p-(i-1)*k]\leftarrow L_{k};\\ \;F\leftarrow F-L_{k};\; \end{array} \end{array}$
8 **end**

#### 2.4.5. L1-norm penalty-based feature selection (L1)

This method introduces a L1-norm regularization term into the objective function to induce the sparsity by shrinking the weights toward zero. Regularization is usually adopted in case that the size of the training set is smaller relative to the dimensionality of the features. This process favorites small parameters of the model to prevent overfitting (Ng, 2004). It is natural in feature selection settings for features with weights of zero to be eliminated from the candidate set. Some researchers have indicated that the L1-norm-based method is better than the L2-norm-based method, especially when there are redundant noise features (Zhu et al., 2004). In this work, we adopted an SVM with an L1-norm penalty to select the important features. The formulation of the objective function is as follows:

(6) $$\underset{\omega_{0},\omega }{\min}\sum_{i=1}^{n}[1-y_{i}(\omega_{0}+\sum_{j=1}^{q}\omega_{j}x_{i,j})]+C\|\omega {\|}_{1}$$

||ω||_1_ is the L1-norm term, in which the ω represents the model weights. The parameter “C” controls the trade-off between the loss and penalty.

### 2.5. Manually operated feature selection

The upper bound of the performance of the proposed features can be verified using the previous automatic feature selection methods. We chose to further evaluate the performance and the importance of the features from different aspects, including different electrodes, locations, rhythms, and feature types. Haufe et al. (2014) indicated that the interpretation of the parameters in backward methods (multivariate classifiers) may lead to the wrong conclusions in neuroimaging data modeling. Hence, in this work, we did not conduct analyses based on the selected features or the corresponding feature weights of the automatic feature selection methods. However, we adopted a simple “searchlight” approach in which we manually selected features from different aspects and evaluated the performances independently.

The performances of automatic and manually operated feature selection were verified by applying a linear SVM. The codes for the data preprocessing, feature extraction, and cross-subject verification processes with different feature selection methods, as well as the extracted features, can be accessed at the following web page: https://github.com/muzixiang/EEG_Emotion_Feature_Engineering.

## 3. Results

### 3.1. Overall evaluation

**Figure 3:**
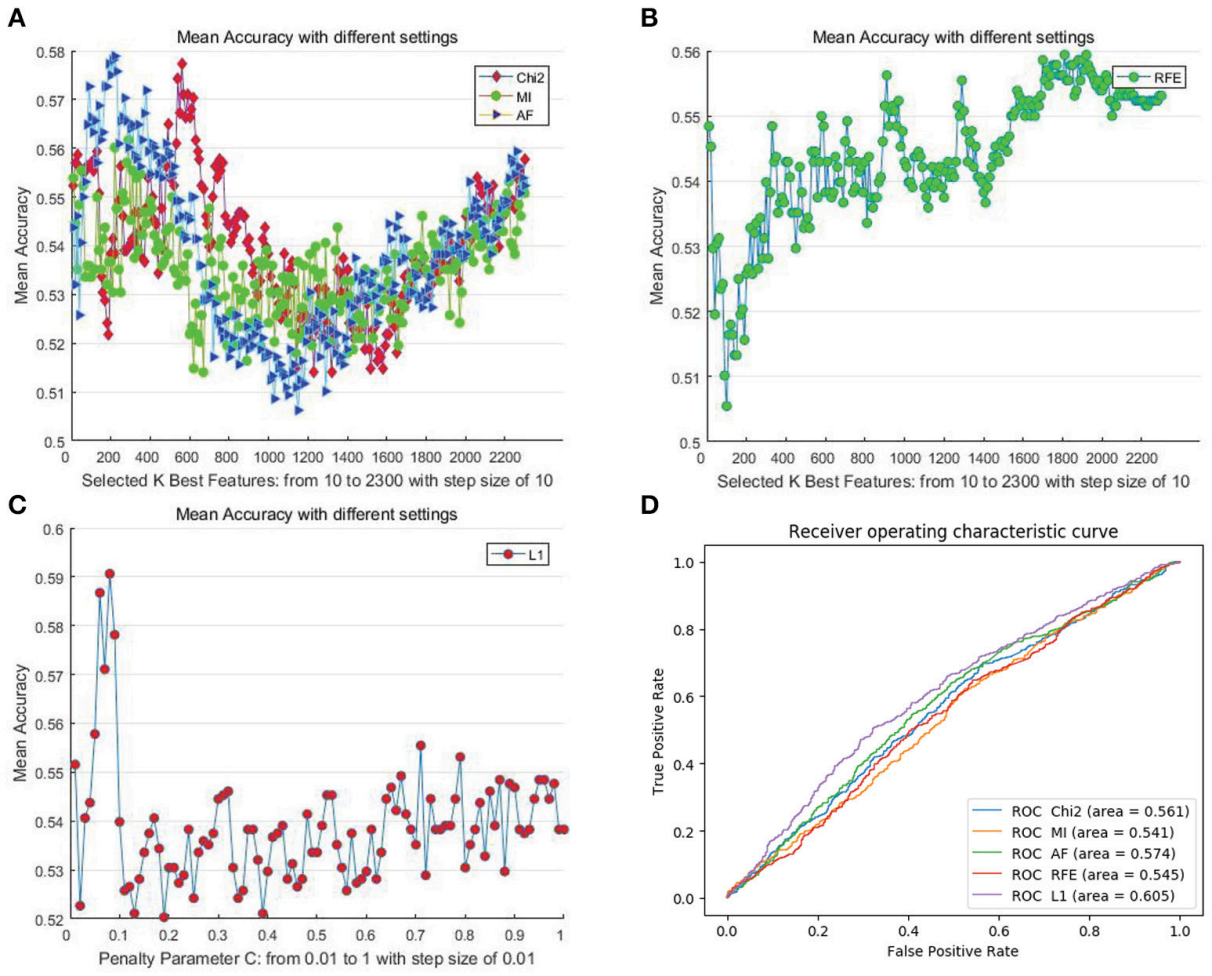
Mean cross-subject recognition performance with different methods and settings on DEAP. **(A)** The filter-based methods. **(B)** The RFE-based method. **(C)** The L1-based method. **(D)** The ROC curves of different methods with their best settings.

**Figure 4:**
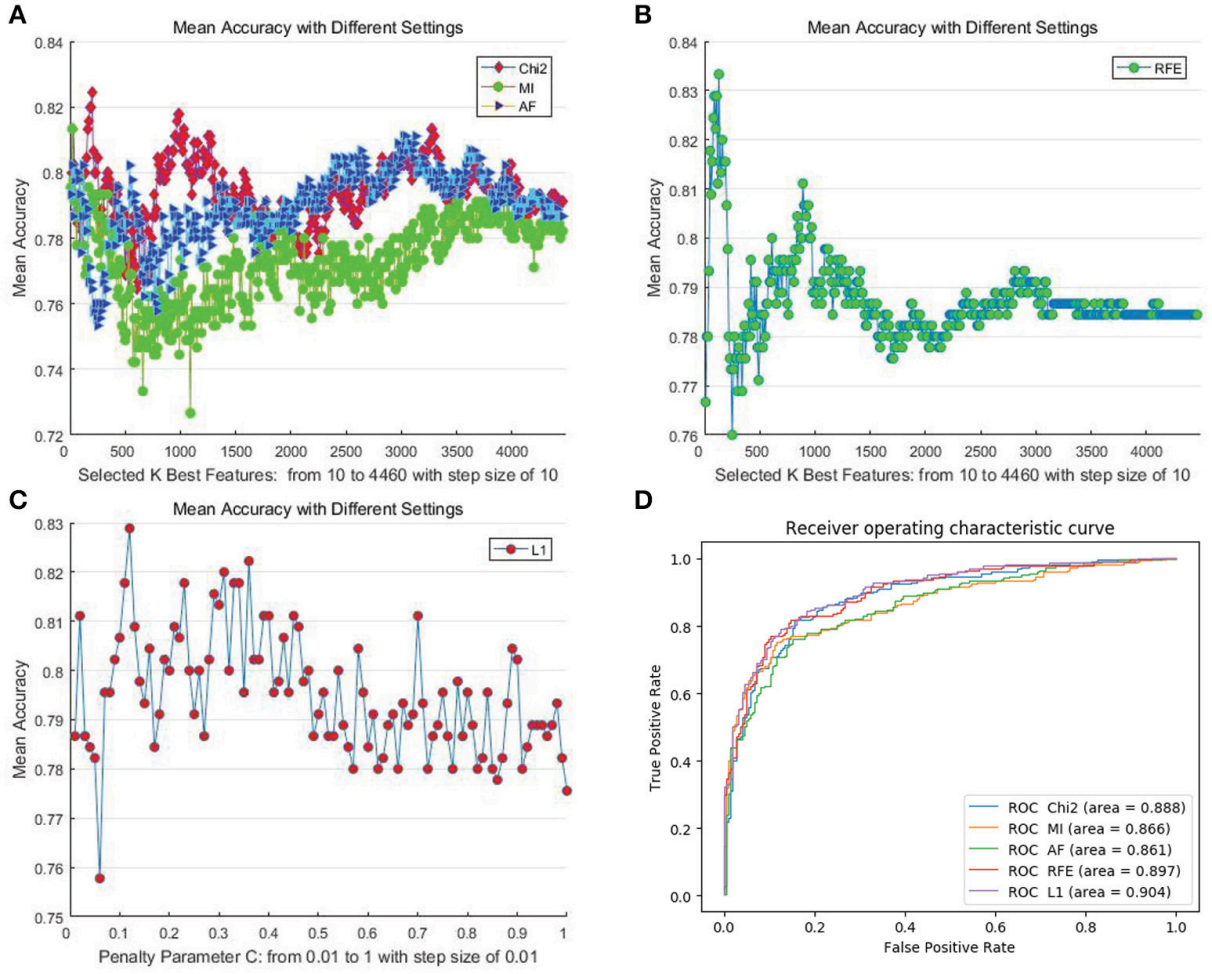
Mean cross-subject recognition performance with different methods and settings on the SEED dataset. **(A)** The filter-based methods. **(B)** The RFE-based method. **(C)** The L1-based method. **(D)** The ROC curves of different methods with their best settings.

We first determined the upper bound of the performance of the proposed features using all of the mentioned automatic feature selection methods. In the experiment, considering the computational overhead of different methods as well as the adequacy of the experiments, we employed different settings for different methods. For filter- and RFE-based methods, we set the step size for the number of selected features to 10. Hence, the number of steps for DEAP and SEED are 230 and 446, respectively. For the L1-norm penalty-based method, we set 100 different values for penalty parameter “C” ranging from 0.01 to 1 with a step size of 0.01. We adopted a “leave-one-subject-out” verification strategy, and the performance was evaluated by the mean recognition accuracy metric. Figures 3A–C, 4A–C illustrate the performance of the automatic feature selection methods with different settings on the DEAP and SEED dataset, respectively.

For DEAP, when no feature selection method was utilized, the recognition performance was 0.5531 (std:0.0839). The best result of 0.5906 (std: 0.0868) was obtained with the L1-norm penalty-based method when the value of “C” is 0.08. For SEED, when no feature selection method was utilized, the recognition performance was 0.7844 (std:0.1119). The best result of 0.8333 (std: 0.1016) was obtained with the RFE-based method when the number of selected features is 130. Table 2 shows the *p*-values calculated through one-way ANOVA test between the method with best performance and other methods. For a better comparison between those methods, as shown in Figures 3D, 4D, we also produced ROC curves. Different feature selection methods were compared by analyzing their ROC curves and the Areas under the ROC curves (AUC). The results showed that the L1-norm penalty-based method outperformed other methods on both DEAP (AUC = 0.605) and SEED (AUC = 0.904). Moreover, the L1-norm penalty-based method incurred a lower computational cost than the other methods. Hence, considering both effectiveness and efficiency, the L1-norm penalty-based feature selection method is recommended to verify the upper bound of the recognition performance when a large amount of features are provided.

**Table 2:** The performance upper bound of the proposed features using different automatic feature selection methods.

**DEAP**	**χ^2^**	**MI**	**AF**	**RFE**
	Step No.: 56	Step No.: 29	Step No.: 22	Step No.: 181
	Mean: 0.5773	Mean: 0.5617	Mean: 0.5789	Mean: 0.5594
	*St.Dev*.: 0.0841	*St.Dev*.: 0.0914	*St.Dev*.: 0.1004	*St.Dev*.: 0.0818
**L1**				
Step No.: 8	*p* = 0.5364	*p* = 0.1992	*p* = 0.6192	*p* = 0.1432
Mean: **0.5906**				
*St.Dev*.: 0.0868				
**SEED**	**χ^2^**	**MI**	**AF**	**L1**
	Step No.: 20	Step No.: 2	Step No.: 2	Step No.: 13
	Mean: 0.8244	Mean: 0.8133	Mean: 0.8111	Mean: 0.8289
	*St.Dev*.: 0.1151	*St.Dev*.: 0.1227	*St.Dev*.: 0.1389	*St.Dev*.: 0.0899
**RFE**				
Step No.: 12	*p* = 0.8242	*p* = 0.6305	*p* = 0.4895	*p* = 0.8999
Mean: **0.8333**				
*St.Dev*.: 0.1016				

*Meanwhile, the p-values calculated through one-way ANOVA between the method with best performance and other methods are also exhibited. The highest mean recognition accuracy is shown in bold type*.

The results demonstrate the effectiveness of our proposed EEG features in cross-subject emotion recognition, especially on the SEED dataset. The performance on DEAP is significantly inferior to that on SEED. This is possibly due to the relatively low quality of the data and the emotional ratings of trials in the DEAP dataset. Hence, we chose to conduct further evaluation only on the SEED dataset.

### 3.2. Evaluation from different perspectives

We also explored the importance of different EEG features in cross-subject recognition from multiple perspectives, including different channels, brain regions, rhythms, and feature types.

**Figure 5:**
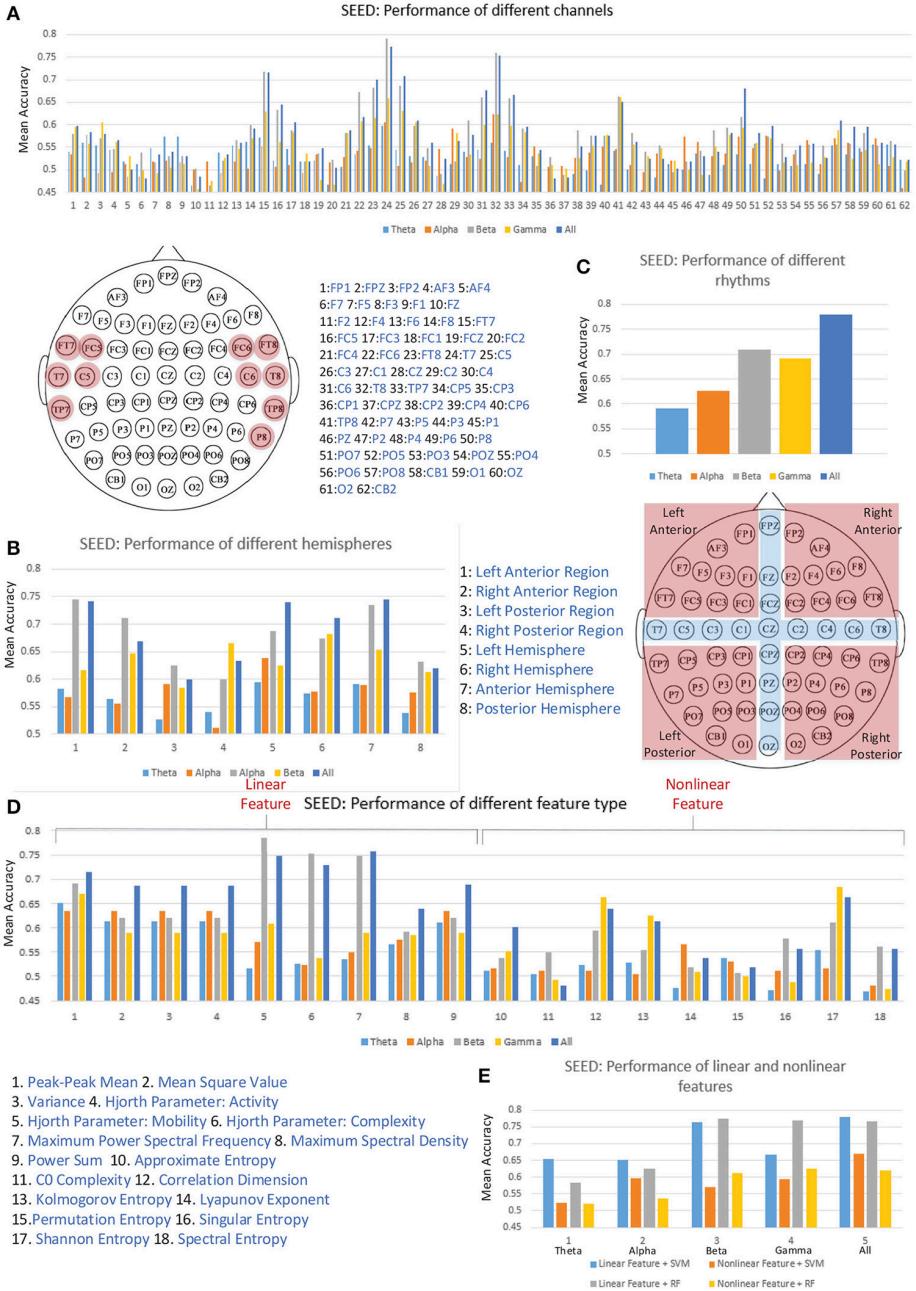
The cross-subject recognition performance based on features from different channels **(A)**, different regions **(B)**, different rhythms **(C)**, different features **(D)**, and different feature types **(E)**.

Figure 5A illustrates the performance of each individual channel. We ranked the performance of these channels and labeled the top one-sixth of the channels on the diagram of the 10–20 international system of electrode placement. The channels on the bilateral temporal regions achieved higher mean accuracies for cross-subject emotion recognition. As shown in Figure 5B, we also evaluated the performance of different regions, including the left-right anterior regions, left-right posterior regions, left-right hemispheres, and anterior-posterior hemispheres. The partition of the regions is illustrated in the figure, and the channels in the cross regions were eliminated when evaluating the performance of the sub-regions. We found that the left anterior region achieved better performance compared to the right anterior region, left posterior region, and the right posterior region, especially when the information in the beta band was utilized. The left hemisphere performed better than the right hemisphere in each band except for the gamma band. Furthermore, the information from the anterior hemisphere enhanced recognition performance in each band more than that from the posterior hemisphere.

Validating the performance of different EEG rhythms was also of interest to us. As we can see in Figure 5C, the individual beta rhythm achieved the best performance, and the higher-frequency beta rhythm and gamma rhythm bands performed better than the lower-frequency theta rhythm and alpha rhythm bands. When the data on all four rhythms were concatenated, the performance was greatly promoted.

The main research objective of this paper is to verify the effectiveness of the proposed features. Thus, we also evaluated the performances of each kind of feature. As shown in Figure 5D, the information on linear features No. 5 (Hjorth parameter: mobility), No. 6 (Hjorth parameter: complexity), and No. 7 (maximum power spectral frequency) in the beta rhythm led to the best mean recognition accuracy. Only the non-linear features No. 12 (correlation dimension), No. 13 (Kolmogorov entropy), and No. 17 (Shannon entropy) can lead to a mean accuracy over 60%. Figure 5E presents the performance comparison between the linear and non-linear features in different frequency bands. The results show that using linear features outperformed the use of non-linear features in each frequency band when linear SVM and random forest (RF) classifiers were applied. Hence, considering the high computational overhead of extracting the non-linear features, solely adopting linear features seems an effective choice for constructing a real-time emotion recognition system. Nevertheless, we should also clarify that the values of the non-linear features calculated in this work may be not optimal. The performance of the non-linear features are influenced by many factors, e.g., the parameter settings and the data volume limitations for the search space. The optimal values of those non-linear features are worth further exploration.

### 3.3. Correlation analysis

**Figure 6:**
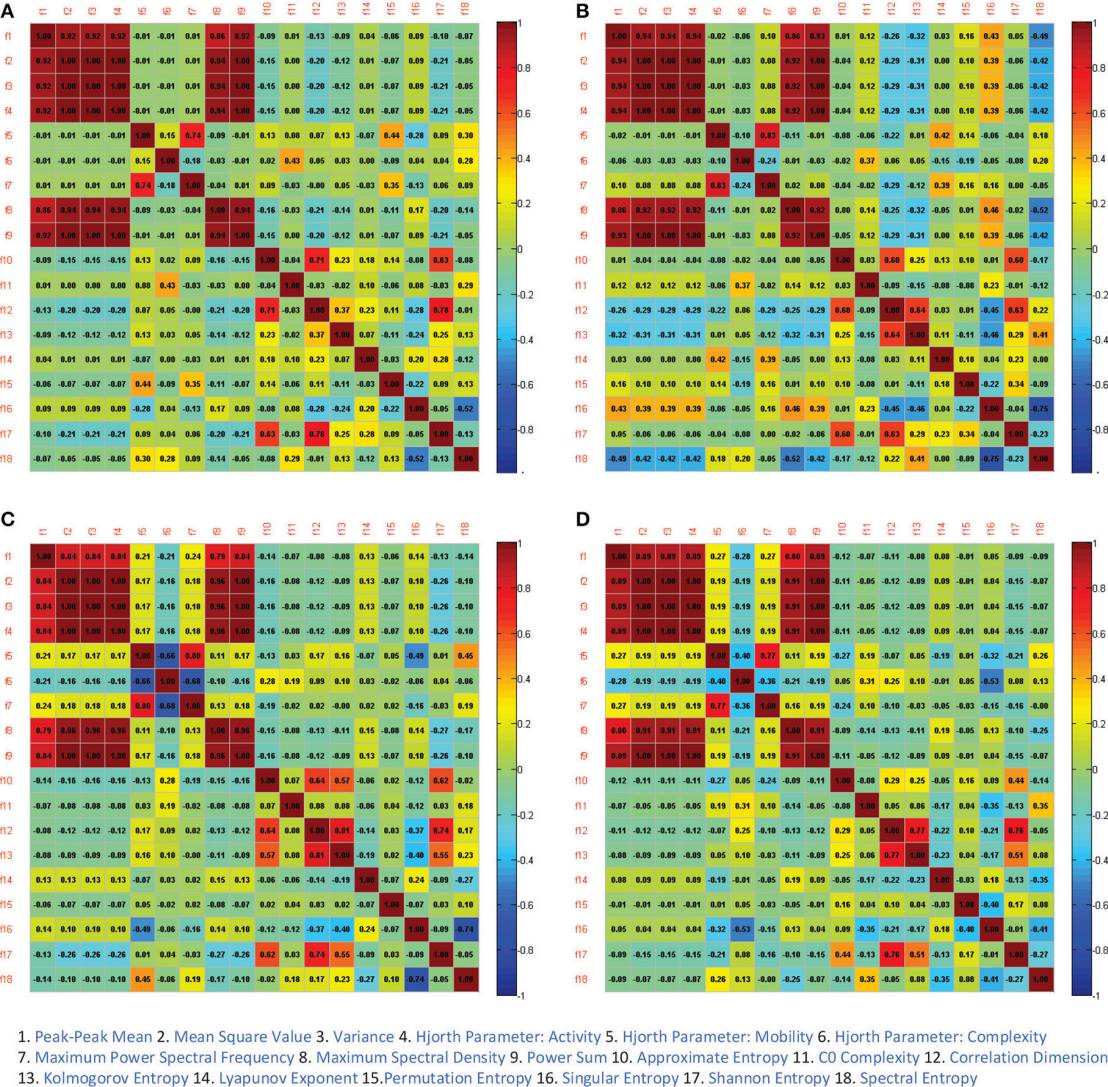
The Pearson correlation between 18 different features (linear features: f1, f2, f3, f4, f5, f6, f7, f8, f9; non-linear features: f10, f11, f12, f13, f14, f15, f16, f17, f18) in theta rhythm **(A)**, alpha rhythm **(B)**, beta rhythm **(C)**, and gamma rhythm **(D)**, respectively.

As we can see in Figure 5D, the performances of some feature type (e.g., features No. 2, No. 3, No. 4, and No. 9) are seemingly identical. This result likely indicates that some features can be highly correlated in identifying a certain emotion class. Hence, for examining those highly correlated features, we calculated the Pearson correlation coefficients for those 18 different feature types. For example, as presented in Figure 6, the linear features No. 2, No. 3, No. 4, and No. 9 are absolutely positively correlated in each rhythm, which explains why the performances of these features are approximately identical. Linear features No. 1 and No. 8 are highly positively correlated with all other linear features except for the Hjorth parameters. For the Hjorth parameters, feature No. 5 is highly positively correlated with feature No. 7 in each rhythm, and is highly negatively correlated with feature No. 6 in the beta rhythm. For the non-linear features, we can see that feature No. 12 is highly and positively correlated with feature No. 13 and No. 17 in the higher-frequency bands, and that feature No. 16 is highly and negatively correlated with feature No. 18 in each rhythm.

**Figure 7:**
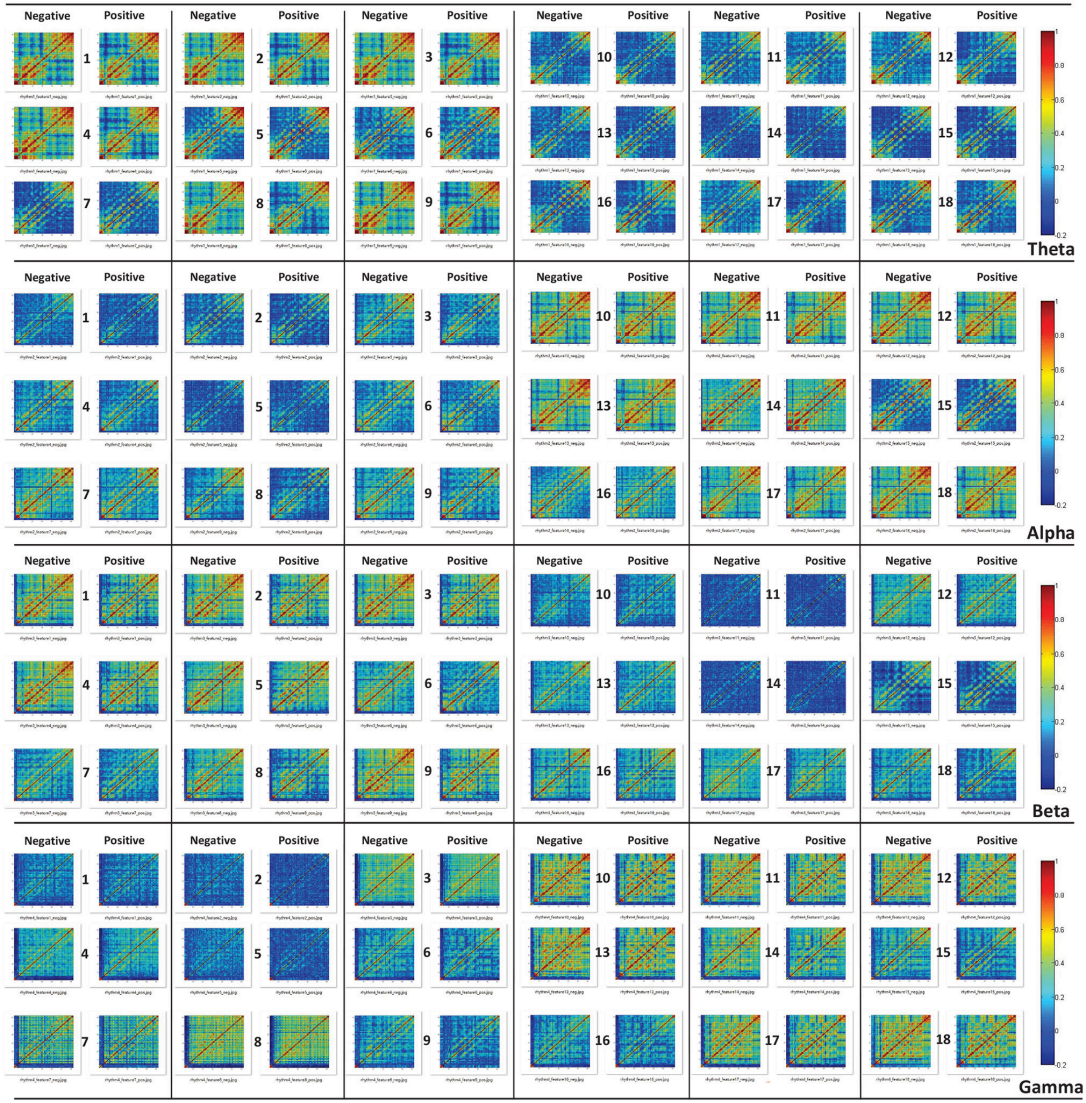
The constructed correlation matrices of the negative emotion group and the positive emotion group when the 18 different features in different rhythms are adopted.

**Figure 8:**
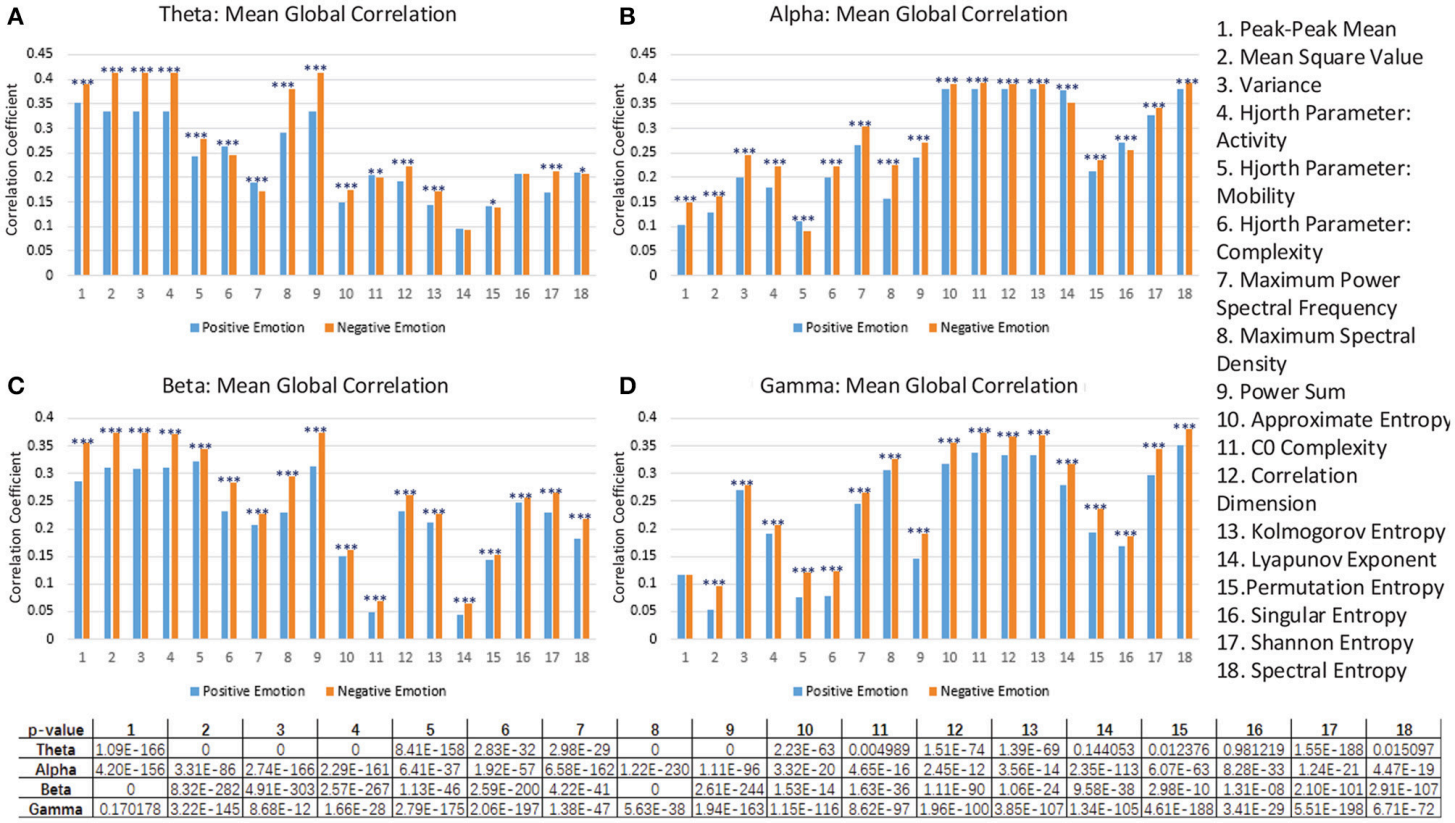
The comparison of the mean global correlation between the groups of negative emotion and positive emotion when the 18 different features in theta rhythm **(A)**, alpha rhythm **(B)**, beta rhythm **(C)**, and gamma rhythm **(D)** are adopted. (***p < 0.001, **p < 0.01, *p < 0.05).

Moreover, through analyzing the correlation of the channels based on those features, we attempted to investigate the underlying mechanisms of those features that allow for differentiating cross-subject emotions. Specifically, for each subject and for each specific feature, we constructed correlation matrices of the 62 channels for subjects' negative trials and positive trials. After all of the correlation matrices had been constructed, we averaged the correlation matrices in the negative group and positive group. The mean correlation matrices for specific features are presented in Figure 7. We also conducted statistical analyses to compare the differences in channel correlations between the negative group and the positive group. The t test results are illustrated in Figure 8. The results in both Figures 7, 8 indicate that for almost every feature, the mean correlations in the negative group are higher than those in the positive group.

The connection network of the 62 channels is represented in the form of a binary matrix, which was constructed based on the obtained correlation matrices. We first needed to determine the threshold of the correlation coefficients, based on which the connection between two channels could be established. To be more specific, the value in the binary matrix was set to 1 when the corresponding value in the correlation matrix was greater than the threshold. Otherwise, the value in the binary matrix was set to 0. The value of 1 in the binary matrix indicates that there is a connection between the two corresponding nodes. Based on the obtained binary matrix, the connection network of the channel nodes was constructed.

**Figure 9:**
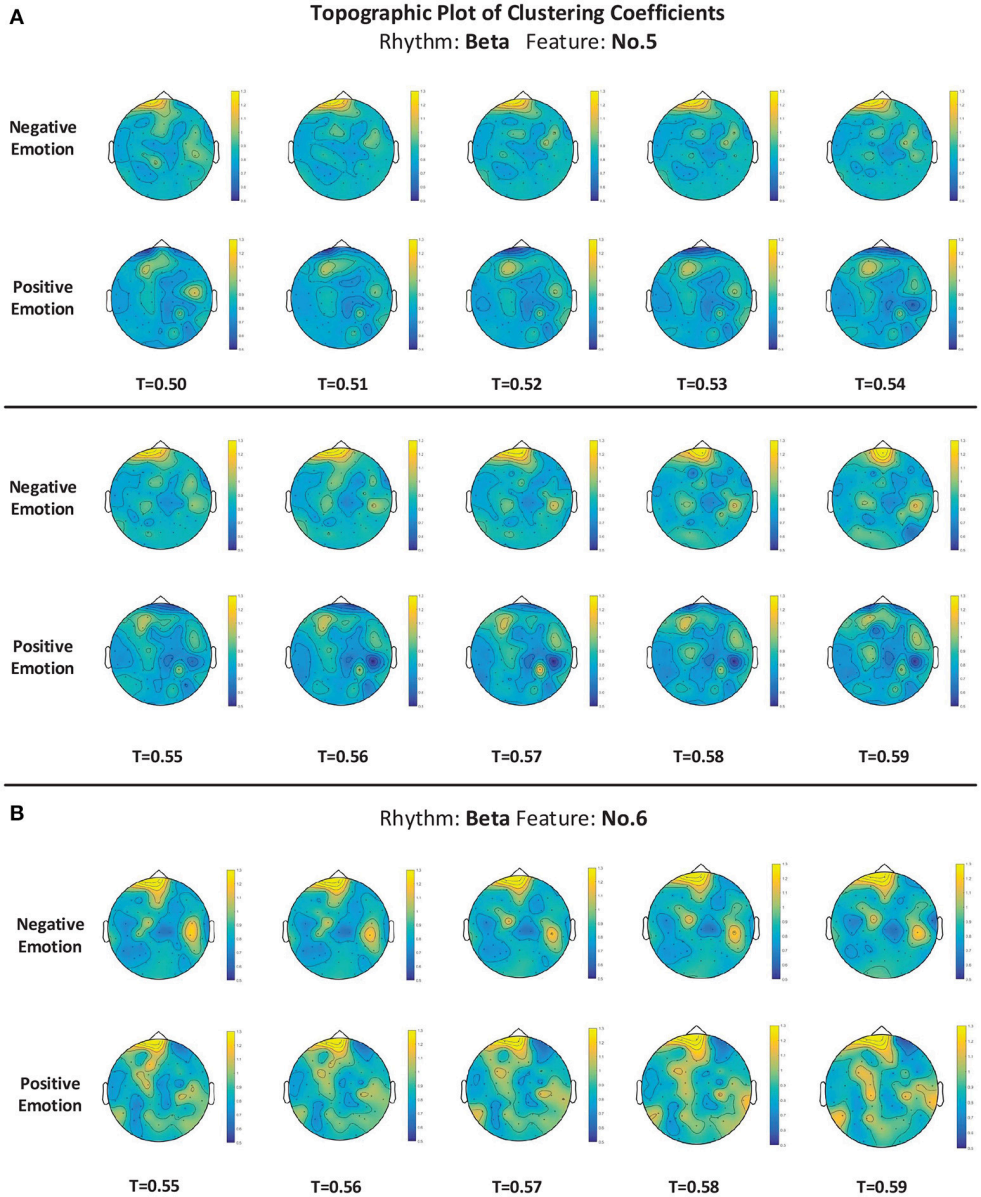
**(A,B)** The topographic plot of the clustering coefficient of the groups of negative emotion and positive emotion when feature No. 5 (Hjorth parameter: mobility) and feature No. 6 (Hjorth parameter: complexity) in beta rhythm were utilized. Conditions with different thresholds (T) are illustrated.

For measuring the coherence of different channel locations in different emotional states, we calculated the clustering coefficients of each node in the connection network. The clustering coefficient was a reflection of the degree of aggregation of different channel locations. The thresholds at which the global clustering coefficients were significantly different between the positive group and negative group are illustrated in Table 3. As mentioned above in Figure 5D, feature No. 5 (Hjorth parameter: mobility) in beta rhythm achieved the best recognition performance. Thus, in this paper, we use this feature as an example to illustrate the topographic plot of the clustering coefficients of the groups with negative and positive emotions. As shown in Figure 9A, at each threshold, the clustering coefficients in the left anterior regions of the negative groups are consistently higher than those of the positive groups. This dynamic may account for the results obtained in Figure 5B indicating that the left anterior regions yields the best recognition performance when beta rhythm information is utilized. Nevertheless, different features and thresholds could have different topographic plots in which the clustering coefficients may be quite different from those of feature No. 5 in the beta rhythm. For example, in addition to feature No. 5 in beta rhythm, feature No. 6 in beta rhythm also led to a high performance. We have also presented the clustering coefficients in Figure 9B. However, the topographic plot was different from that in Figure 9A, and the left anterior region was no longer significantly different between the two groups. Hence, the important locations for emotion recognition cannot be determined simply by analyzing only one or two features.

**Table 3:** The threshold scope that can significantly differentiate the clustering coefficients in groups of positive emotion and negative emotion (*p* < 0.05).

**Rhythm**	**Feature**	**Threshold scope**	**Feature**	**Threshold scope**
Theta	No.1	0.34~0.65, 0.92~0.99	No.10	0.01~0.36, 0.62~0.63, 0.69~0.71, 0.89~0.92
Theta	No.2	0.01~0.66, 0.92~0.99	No.11	0.14~0.47, 0.71~0.74, 0.84~0.87, 0.97~0.98
Theta	No.3	0.01~0.66, 0.92~0.99	No.12	0.01~0.35, 0.59~0.62, 0.77~0.78, 0.94~0.96
Theta	No.4	0.01~0.66, 0.92~0.99	No.13	0.01~0.20, 0.49~0.50
Theta	No.5	0.01~0.39	No.14	0.21~0.31
Theta	No.6	0.01~0.24	No.15	0.07~0.32, 0.40~0.49, 0.70~0.72, 0.95~0.96
Theta	No.7	0.01~0.15	No.16	0.01~0.34, 0.54~0.58
Theta	No.8	0.01~0.65, 0.93~0.99	No.17	0.01~0.42
Theta	No.9	0.01~0.66, 0.93~0.99	No.18	0.49~0.50, 0.90~0.98
Alpha	No.1	0.01~0.58, 0.66~0.95	No.10	0.01~0.08, 0.53~0.56, 0.67~0.72, 0.96~0.99
Alpha	No.2	0.01~0.27, 0.49~0.60, 0.75~0.91	No.11	0.01~0.32, 0.64~0.76, 0.95~0.99
Alpha	No.3	0.01~0.41, 0.56~0.66, 0.83~0.95	No.12	0.01~0.08, 0.23~0.32, 0.67~0.76, 0.95~0.99
Alpha	No.4	0.01~0.40, 0.47~0.59, 0.90~0.93	No.13	0.01~0.05, 0.28~0.32, 0.67~0.78, 0.95~0.99
Alpha	No.5	0.01~0.29, 0.50~0.52	No.14	0.01~0.25
Alpha	No.6	0.01~0.25, 0.48~0.73	No.15	0.01~0.17, 0.24~0.28, 0.38~0.40, 0.89~0.92
Alpha	No.7	0.01~0.35, 0.46~0.48, 0.95~0.97	No.16	0.01~0.14, 0.36~0.39
Alpha	No.8	0.01~0.36, 0.49~0.70, 0.79~0.99	No.17	0.01~0.05, 0.64~0.66, 0.90~0.99
Alpha	No.9	0.01~0.35, 0.82~0.85, 0.95~0.98	No.18	0.01~0.06, 0.65~0.78, 0.95~0.99
Beta	No.1	0.01~0.44, 0.53~0.58, 0.90~0.92	No.10	0.01~0.13, 0.68~0.69
Beta	No.2	0.01~0.47	No.11	0.01~0.32, 0.42~0.43, 0.46~0.54, 0.67~0.80
Beta	No.3	0.01~0.49, 0.85~0.86	No.12	0.01~0.26, 0.91~0.98
Beta	No.4	0.01~0.49, 0.53~0.56, 0.85~0.86	No.13	0.01~0.34, 0.48~0.54, 0.87~0.92
Beta	No.5	0.01~0.16, 0.20~0.23, 0.48~0.61	No.14	0.01~0.26, 0.38~0.43, 0.62~0.74, 0.81~0.87
Beta	No.6	0.01~0.41, 0.55~0.64, 0.85~0.91	No.15	0.01~0.21
Beta	No.7	0.01~0.31, 0.67~0.71, 0.91~0.99	No.16	0.01~0.21, 0.57~0.62, 0.81~0.83, 0.86~0.88
Beta	No.8	0.01~0.40, 0.86~0.89, 0.96~0.99	No.17	0.01~0.12, 0.67~0.69, 0.86~0.88, 0.95~0.99
Beta	No.9	0.01~0.47	No.18	0.01~0.29, 0.39~0.46, 0.74~0.88
Gamma	No.1	0.14~0.27, 0.66~0.70, 0.76~0.81	No.10	0.15~0.33, 0.40~0.64, 0.83~0.88
Gamma	No.2	0.01~0.33, 0.85~0.98	No.11	0.17~0.22, 0.28~0.29
Gamma	No.3	0.01~0.17, 0.50~0.51	No.12	0.26~0.49
Gamma	No.4	0.01~0.47, 0.97~0.98	No.13	0.23~0.54
Gamma	No.5	0.01~0.31, 0.73~0.98	No.14	0.01~0.35, 0.97~0.99
Gamma	No.6	0.01~0.30, 0.80~0.99	No.15	0.01~0.22, 0.42~0.47, 0.72~0.74, 0.83~0.88
Gamma	No.7	0.01~0.35, 0.93~0.99	No.16	0.01~0.05, 0.81~0.84
Gamma	No.8	0.48~0.61, 0.98~0.99	No.17	0.01~0.49, 0.97~0.99
Gamma	No.9	0.01~0.32, 0.90~0.91, 0.95~0.99	No.18	0.01~0.17, 0.21~0.32, 0.40~0.42

As described above, we should point out that such correlation analysis may not be adequate to fully interpret the mechanism of the features. Moreover, for example, as shown in Figure 8, the feature No. 1 in the gamma rhythm cannot significantly differentiate the correlation coefficients of the two groups. However, as shown in Figure 5D, this feature can still lead to a better performance than most of the non-linear features.

## 4. Discussions

In this work, we verified the effectiveness of 18 kinds of EEG features in cross-subject emotion recognition, including 9 kinds of time-frequency domain features and 9 kinds of dynamical system features. We adopted a “leave-one-subject-out” method to verify the performance of the proposed features. After automatic feature selection, the highest mean recognition accuracies of 59.06% (AUC = 0.605) on the DEAP dataset and 83.33% (AUC = 0.904) on the SEED dataset were reached. The performance on DEAP was not as good as that on SEED, which could be due to the low quality of the data in the emotional ratings of the trials. The noise in the emotional ratings degraded the ability of the model to differentiate between different classes. Through drawing the ROC curves, we found that the L1-norm penalty-based feature selection method exhibited robust performance on both two datasets. Considering its lower computational overhead, this method is the best strategy to adopt when analyzing large numbers of candidate features.

We also evaluated the cross-subject recognition performance from different perspectives, including different EEG channels, different regions, different rhythms, different features, and different feature types. We chose to conduct analyses on the SEED dataset because of its better performance. Specifically, through evaluation over individual channels, we found that the channels with the best performances were mainly located in the bilateral temporal regions, which was consistent with the finding in Soleymani et al. (2012), Zheng et al. (2016). We partitioned the channels into different groups according to the different regions and evaluated the performances of the different groups. We found that the left anterior region achieved a better performance compared to the other sub-regions, especially when the information in the beta band was utilized. The left hemisphere performed better than the right hemisphere except for in the gamma band. Furthermore, the anterior hemisphere exhibited an improved recognition performance compared to the posterior hemisphere, especially when data from all rhythms were utilized. The relationship between emotion recognition and frontal regions was illustrated in the studies of Schmidt and Trainor (2001), Lin et al. (2010). Schmidt and Trainor (2001) found the relatively higher left frontal EEG activity under exposure to happy musical excerpts and relatively higher right frontal EEG activity under exposure to sad musical excerpts, and the overall frontal EEG activity could distinguish the intensity of the emotions. Lin et al. (2010) found that the frontal and parietal electrode pairs were the most informative on emotional states.

The evaluation of different rhythms indicated that the information in higher-frequency bands contributed more to cross-subject emotion recognition compared to lower-frequency bands. The effectiveness of the beta and gamma rhythms in promoting emotion recognition was also presented in Lin et al. (2010), Soleymani et al. (2012), Zheng et al. (2016). Moreover, some neuroscience studies have found that emotion-related neural information mainly resides in higher-frequency bands (Müller et al., 1999; Kortelainen et al., 2015). By evaluating the performances of individual features, we found that linear features No. 5 (Hjorth parameter: mobility), No. 6 (Hjorth parameter: complexity), and No. 7 (maximum power spectral frequency) in the beta rhythm led to the best mean recognition accuracy. However, the Hjorth parameters have not been widely adopted in EEG-based emotion recognition. We also found that the combination of the linear features greatly outperformed the combination of non-linear features in each frequency band. Considering the high computational overhead in extracting the non-linear features, adopting linear features in designing real-time emotion recognition systems is recommended. Nevertheless, the non-linear features calculated here may be not the optimal ones, given that emotional information is very likely processed in a non-linear way. The optimal values of the non-linear features for reflecting emotional processes are certainly worth further exploration.

For a better understanding of the mechanisms of those features that allow for differentiating between emotions, we further conducted a correlation analysis for 62 channels for each feature. We calculated and constructed correlation matrices using different features. We found that for nearly every feature, the negative group has a higher mean correlation coefficient than the positive group. Based on the constructed correlation matrices, we further calculated the clustering coefficients at different thresholds. We listed the thresholds at which the clustering coefficients were significantly different between the two groups, and we presented the clustering coefficients in detail with a topographic plot for features No. 5 and No. 6 in the beta rhythm. The preliminary analysis implied that the features' ability to reflect the channel correlation may contribute to the recognition of emotions. Nevertheless, considering the differences in the clustering coefficients of the different features, we should note that the correlation analysis is not sufficient to fully explain the mechanism of those features or to determine the important locations. Additional analyses from different perspectives using different approaches are still needed in future work.

In the future, we should also further study the oscillatory and temporal process of emotion perception based on these features and verify the effectiveness of the proposed features on other datasets. In addition to correlation analysis, we need an in-depth study of the mechanisms of the features that allow for differentiating between positive and negative emotions. Another potential line of research is to further verify the ability of those features to identify emotion-related mental disorders, e.g., depression, as well as the effectiveness of those features in studying other cognitive processes.

## Author contributions

XL proposed the idea, conducted the experiments, and wrote the manuscript. DS, PZ, YZ, and YH provided advice on the research approaches, guided the experiments, and checked and revised the manuscript. BH offered important help on EEG processing and analysis methods.

### Conflict of interest statement

The authors declare that the research was conducted in the absence of any commercial or financial relationships that could be construed as a potential conflict of interest.
